# Bromidotricarbon­yl[4-chloro-*N*-(2-pyridyl­methyl­idene)aniline-κ^2^ *N*,*N*′]rhenium(I)

**DOI:** 10.1107/S1600536810044211

**Published:** 2010-11-24

**Authors:** Mehdi Khalaj, Saeed Dehghanpour, Roghaieh Aleeshah, Ali Mahmoudi

**Affiliations:** a Department of Chemistry, Islamic Azad University, Buinzahra Branch, Buinzahra, Qazvin, Iran; b Department of Chemistry, Alzahra University, Vanak, Tehran, Iran; c Department of Chemistry, Islamic Azad University, Karaj Branch, Karaj, Iran

## Abstract

In the title compound, [ReBr(C_12_H_9_ClN_2_)(CO)_3_], the Re^I^ atom has a distorted octa­hedral configuration with the three carbonyl ligands showing a facial arrangement. The main distortion of the octa­hedron is due to a small bite angle of the chelating bidentate diimine ligand [N—Re—N = 75.3 (3)°].

## Related literature

For the synthesis of (4-chloro­phen­yl)pyridin-2-yl­methyl­ene­amine, see: Dehghanpour & Mahmoudi (2007 ▶). For related structures, see: Dehghanpour *et al.* (2009 ▶, 2010 ▶); Dehghanpour & Mahmoudi (2010 ▶)
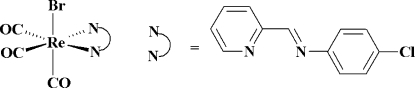


## Experimental

### 

#### Crystal data

[ReBr(C_12_H_9_ClN_2_)(CO)_3_]
*M* *_r_* = 566.80
Triclinic, 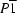

*a* = 8.6559 (8) Å
*b* = 8.9037 (8) Å
*c* = 10.9442 (9) Å
α = 75.691 (5)°
β = 83.001 (5)°
γ = 81.808 (5)°
*V* = 805.65 (12) Å^3^
*Z* = 2
Mo *K*α radiation
μ = 10.20 mm^−1^
*T* = 150 K
0.10 × 0.09 × 0.03 mm

#### Data collection

Nonius KappaCCD diffractometer
Absorption correction: multi-scan (*SORTAV*; Blessing, 1995 ▶) *T*_min_ = 0.425, *T*_max_ = 0.734
8206 measured reflections
3651 independent reflections
2722 reflections with *I* > 2σ(*I*)
*R*_int_ = 0.077

#### Refinement

*R*[*F* ^2^ > 2σ(*F* ^2^)] = 0.052
*wR*(*F* ^2^) = 0.126
*S* = 1.03
3651 reflections
208 parameters
H-atom parameters constrained
Δρ_max_ = 3.45 e Å^−3^
Δρ_min_ = −2.54 e Å^−3^

### 

Data collection: *COLLECT* (Nonius, 2002 ▶); cell refinement: *DENZO*–*SMN* (Otwinowski & Minor, 1997 ▶); data reduction: *DENZO*–*SMN*; program(s) used to solve structure: *SIR92* (Altomare *et al.*, 1994 ▶); program(s) used to refine structure: *SHELXL97* (Sheldrick, 2008 ▶); molecular graphics: *PLATON* (Spek, 2009 ▶); software used to prepare material for publication: *SHELXL97*.

## Supplementary Material

Crystal structure: contains datablocks I, global. DOI: 10.1107/S1600536810044211/gk2300sup1.cif

Structure factors: contains datablocks I. DOI: 10.1107/S1600536810044211/gk2300Isup2.hkl

Additional supplementary materials:  crystallographic information; 3D view; checkCIF report

## Figures and Tables

**Table 1:** Selected bond lengths (Å)

Re1—C2	1.919 (11)
Re1—C3	1.937 (10)
Re1—C1	1.969 (13)
Re1—N1	2.170 (8)
Re1—N2	2.182 (8)
Re1—Br1	2.6165 (11)

**Fig. 1.**
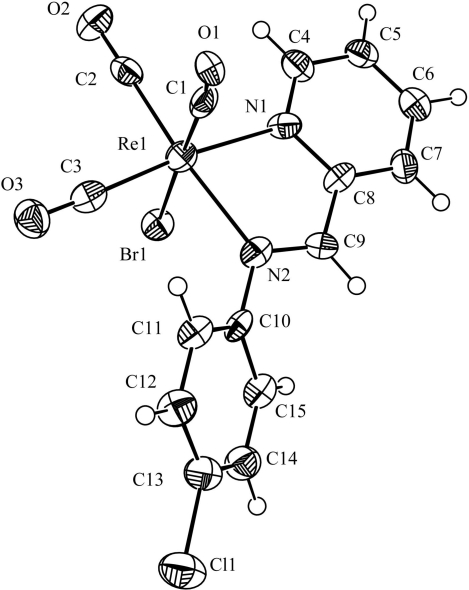
A view of the structure of the title complex, with displacement ellipsoids drawn at the 50% probability level. H atoms are represented as spheres of arbitrary radius.

## supplementary crystallographic information

### Comment

The title complex, (I), (Fig. 1) was prepared by the reaction of Re(CO)_5_Br with the bidentate ligand (4-chlorophenyl)pyridin-2-ylmethyleneamine.

The Re center in (I) has a distorted octahedral geometry, with the three carbonyls arranged in a facial configuration as expected for d^6^ ReCO_3_^+^ compounds. The diimine ligand binds to the metal in a bidentate fashion through the nitrogen atoms. The last site in the coordination sphere is occupied by bromide. The C–O bonds of the carbonyls are typical for ReCO_3_^+^ complexes with the bond lengths in the range of 1.058 (12)–1.135 (11) Å (Dehghanpour *et al.*, 2009; Dehghanpour *et al.*, 2010). The Re–C bonds have standard lengths, with ranges of 1.919 (11)–1.969 (13) Å, and the Re–*X* bond is as expected for these complexes. The metal–nitrogen bonds of the diimine have bond lengths of 2.170 (8) and 2.182 (8) Å. The steric requirements of the bidentate ligands cause distortion of the octahedral coordination which is most clearly seen for the ligand bite angle (N1–Re1–N2, 75.3 (3)°). The coordinated bromide is slightly titled toward the diimine ligand, causing a narrowing of the N–Re–Br angles (*e.g.* 84.6 (2)° for N1–Re1–Br1, 82.3 (2)° for N2–Re1–Br1).

### Experimental

A mixture of [Re(CO)_5_Br] (406 mg, 1 mmol) and (4-chlorophenyl)pyridin-2-ylmethyleneamine (216 mg, 1 mmol) in dry, degassed toluene (30 ml) was heated to reflux for 4 h under N_2_ to give a bright red solution. The solvent was removed under vacuum and the crude material recrystallized from CH_2_Cl_2_/hexane to give [Re(CO)_3_Br(C_12_H_9_ClN_2_)] as pure red crystals. Yield: 91%. Calc. for C_15_H_9_ClBrN_2_O_3_Re: C 31.79, H 1.59, N 4.94%; found: C 31.89, H 1.50, N 4.99%.

### Refinement

All H atoms were positioned geometrically and their parameters refined in a riding model approximatiom with *U*_iso_(H)=1.2 *U*_eq_(C) . The highest density peak in the final differnce Fourier map is 3.45eÅ^-3^ and is located 1.86Å from C2 while the deepest hole is -2.45eÅ^-3^ and is located 0.88Å from Re1. These effects may be caused by the fairly low redundancy of the reflections used for the absorption correction. There is also a possibility that there is some whole molecule disorder present but this could not be identified  as it was in a related crystal structure (Dehghanpour & Mahmoudi, 2010).

### Crystal data

**Table:**

[ReBr(C_12_H_9_ClN_2_)(CO)_3_]	*Z* = 2
*M**_r_* = 566.80	*F*(000) = 528
Triclinic, *P*1	*D*_x_ = 2.336 Mg m^−^^3^
Hall symbol: -P 1	Mo *K*α radiation, λ = 0.71073 Å
*a* = 8.6559 (8) Å	Cell parameters from 8206 reflections
*b* = 8.9037 (8) Å	θ = 2.7–27.6°
*c* = 10.9442 (9) Å	µ = 10.20 mm^−^^1^
α = 75.691 (5)°	*T* = 150 K
β = 83.001 (5)°	Plate, orange
γ = 81.808 (5)°	0.10 × 0.09 × 0.03 mm
*V* = 805.65 (12) Å^3^	

### Data collection

**Table:**

Nonius KappaCCD diffractometer	3651 independent reflections
Radiation source: fine-focus sealed tube	2722 reflections with *I* > 2σ(*I*)
graphite	*R*_int_ = 0.077
Detector resolution: 9 pixels mm^-1^	θ_max_ = 27.6°, θ_min_ = 2.7°
φ scans and ω scans with κ offsets	*h* = −11→11
Absorption correction: multi-scan (*SORTAV*; Blessing, 1995)	*k* = −11→11
*T*_min_ = 0.425, *T*_max_ = 0.734	*l* = −12→14
8206 measured reflections	

### Refinement

**Table:**

Refinement on *F*^2^	Primary atom site location: structure-invariant direct methods
Least-squares matrix: full	Secondary atom site location: difference Fourier map
*R*[*F*^2^ > 2σ(*F*^2^)] = 0.052	Hydrogen site location: inferred from neighbouring sites
*wR*(*F*^2^) = 0.126	H-atom parameters constrained
*S* = 1.03	*w* = 1/[σ^2^(*F*_o_^2^) + (0.0437*P*)^2^ + 4.4525*P*] where *P* = (*F*_o_^2^ + 2*F*_c_^2^)/3
3651 reflections	(Δ/σ)_max_ = 0.001
208 parameters	Δρ_max_ = 3.45 e Å^−^^3^
0 restraints	Δρ_min_ = −2.54 e Å^−^^3^

### Special details

**Table:**

Geometry. All e.s.d.'s (except the e.s.d. in the dihedral angle between two l.s. planes) are estimated using the full covariance matrix. The cell e.s.d.'s are taken into account individually in the estimation of e.s.d.'s in distances, angles and torsion angles; correlations between e.s.d.'s in cell parameters are only used when they are defined by crystal symmetry. An approximate (isotropic) treatment of cell e.s.d.'s is used for estimating e.s.d.'s involving l.s. planes.
Refinement. Refinement of *F*^2^ against ALL reflections. The weighted *R*-factor *wR* and goodness of fit *S* are based on *F*^2^, conventional *R*-factors *R* are based on *F*, with *F* set to zero for negative *F*^2^. The threshold expression of *F*^2^ > σ(*F*^2^) is used only for calculating *R*-factors(gt) *etc*. and is not relevant to the choice of reflections for refinement. *R*-factors based on *F*^2^ are statistically about twice as large as those based on *F*, and *R*- factors based on ALL data will be even larger.

### Fractional atomic coordinates and isotropic or equivalent isotropic displacement parameters (Å^2^)

**Table:**

	*x*	*y*	*z*	*U*_iso_*/*U*_eq_	
Re1	0.72202 (5)	0.92191 (4)	0.74692 (4)	0.03208 (15)	
Br1	0.79387 (12)	0.78337 (11)	0.97638 (9)	0.0352 (2)	
Cl1	1.3685 (3)	0.3425 (4)	0.5681 (3)	0.0536 (7)	
O1	0.6432 (7)	1.0842 (8)	0.4811 (7)	0.0348 (16)	
O2	0.6196 (9)	1.2317 (8)	0.8222 (7)	0.0454 (19)	
O3	1.0512 (9)	1.0234 (8)	0.6675 (7)	0.0440 (18)	
N1	0.5004 (9)	0.8272 (9)	0.8084 (7)	0.0324 (19)	
N2	0.7661 (10)	0.6836 (9)	0.7214 (7)	0.0299 (18)	
C1	0.6666 (12)	1.0207 (11)	0.5736 (11)	0.034 (2)	
C2	0.6572 (11)	1.1172 (12)	0.7930 (9)	0.034 (2)	
C3	0.9309 (13)	0.9829 (11)	0.6976 (9)	0.034 (2)	
C4	0.3679 (12)	0.8976 (13)	0.8541 (9)	0.038 (2)	
H4A	0.3654	1.0036	0.8574	0.045*	
C5	0.2312 (12)	0.8245 (12)	0.8979 (9)	0.035 (2)	
H5A	0.1401	0.8779	0.9327	0.042*	
C6	0.2341 (12)	0.6726 (12)	0.8883 (9)	0.035 (2)	
H6A	0.1433	0.6199	0.9141	0.042*	
C7	0.3699 (12)	0.5983 (12)	0.8409 (10)	0.039 (2)	
H7A	0.3730	0.4931	0.8354	0.046*	
C8	0.5019 (12)	0.6742 (10)	0.8011 (9)	0.032 (2)	
C9	0.6501 (11)	0.6024 (11)	0.7544 (9)	0.034 (2)	
H9A	0.6616	0.4968	0.7481	0.041*	
C10	0.9115 (12)	0.6014 (10)	0.6875 (9)	0.031 (2)	
C11	1.0098 (12)	0.6759 (11)	0.5850 (9)	0.034 (2)	
H11A	0.9797	0.7799	0.5405	0.041*	
C12	1.1509 (13)	0.5960 (12)	0.5499 (10)	0.041 (3)	
H12A	1.2183	0.6454	0.4811	0.050*	
C13	1.1937 (12)	0.4441 (12)	0.6150 (9)	0.038 (2)	
C14	1.1010 (13)	0.3726 (13)	0.7181 (10)	0.041 (3)	
H14A	1.1341	0.2701	0.7641	0.049*	
C15	0.9604 (12)	0.4498 (11)	0.7544 (9)	0.035 (2)	
H15A	0.8961	0.4000	0.8252	0.043*	

### Atomic displacement parameters (Å^2^)

**Table:**

	*U*^11^	*U*^22^	*U*^33^	*U*^12^	*U*^13^	*U*^23^
Re1	0.0359 (3)	0.0276 (2)	0.0315 (2)	−0.00496 (16)	−0.00217 (17)	−0.00434 (16)
Br1	0.0400 (6)	0.0342 (5)	0.0299 (5)	−0.0039 (4)	−0.0040 (4)	−0.0047 (4)
Cl1	0.0439 (16)	0.0624 (18)	0.0549 (17)	0.0102 (14)	−0.0067 (14)	−0.0230 (15)
O1	0.022 (4)	0.037 (4)	0.047 (4)	−0.007 (3)	−0.003 (3)	−0.010 (4)
O2	0.056 (5)	0.031 (4)	0.050 (5)	−0.006 (4)	0.000 (4)	−0.014 (3)
O3	0.038 (4)	0.046 (4)	0.045 (4)	−0.010 (4)	−0.004 (4)	−0.002 (3)
N1	0.031 (4)	0.027 (4)	0.035 (4)	0.003 (3)	0.001 (4)	−0.005 (3)
N2	0.041 (5)	0.026 (4)	0.023 (4)	−0.003 (4)	−0.005 (4)	−0.006 (3)
C1	0.028 (5)	0.026 (5)	0.053 (7)	−0.008 (4)	0.011 (5)	−0.022 (5)
C2	0.022 (5)	0.039 (6)	0.038 (6)	0.000 (4)	−0.002 (4)	−0.007 (5)
C3	0.042 (6)	0.030 (5)	0.027 (5)	−0.001 (5)	0.000 (5)	−0.007 (4)
C4	0.030 (6)	0.046 (6)	0.038 (6)	0.000 (5)	−0.007 (5)	−0.014 (5)
C5	0.032 (6)	0.039 (6)	0.034 (5)	0.004 (4)	−0.008 (4)	−0.010 (4)
C6	0.032 (6)	0.044 (6)	0.028 (5)	−0.006 (5)	−0.003 (4)	−0.005 (4)
C7	0.037 (6)	0.035 (5)	0.045 (6)	−0.011 (5)	−0.014 (5)	−0.004 (5)
C8	0.043 (6)	0.022 (4)	0.033 (5)	−0.004 (4)	0.000 (5)	−0.008 (4)
C9	0.030 (5)	0.029 (5)	0.042 (6)	0.004 (4)	−0.009 (4)	−0.007 (4)
C10	0.041 (6)	0.023 (5)	0.035 (5)	−0.006 (4)	−0.004 (5)	−0.016 (4)
C11	0.044 (6)	0.026 (5)	0.032 (5)	−0.003 (4)	−0.003 (5)	−0.007 (4)
C12	0.046 (7)	0.038 (6)	0.039 (6)	−0.003 (5)	−0.001 (5)	−0.009 (5)
C13	0.039 (6)	0.045 (6)	0.033 (5)	0.004 (5)	−0.010 (5)	−0.017 (5)
C14	0.043 (6)	0.039 (6)	0.044 (6)	0.000 (5)	−0.009 (5)	−0.014 (5)
C15	0.043 (6)	0.031 (5)	0.034 (5)	−0.007 (5)	−0.002 (5)	−0.008 (4)

### Geometric parameters (Å, °)

**Table:**

Re1—C2	1.919 (11)	C5—H5A	0.9500
Re1—C3	1.937 (10)	C6—C7	1.374 (14)
Re1—C1	1.969 (13)	C6—H6A	0.9500
Re1—N1	2.170 (8)	C7—C8	1.380 (13)
Re1—N2	2.182 (8)	C7—H7A	0.9500
Re1—Br1	2.6165 (11)	C8—C9	1.443 (13)
Cl1—C13	1.738 (11)	C9—H9A	0.9500
O1—C1	1.058 (12)	C10—C15	1.400 (13)
O2—C2	1.135 (12)	C10—C11	1.409 (13)
O3—C3	1.135 (11)	C11—C12	1.385 (14)
N1—C4	1.330 (12)	C11—H11A	0.9500
N1—C8	1.382 (11)	C12—C13	1.386 (14)
N2—C9	1.285 (12)	C12—H12A	0.9500
N2—C10	1.419 (12)	C13—C14	1.375 (14)
C4—C5	1.408 (13)	C14—C15	1.375 (14)
C4—H4A	0.9500	C14—H14A	0.9500
C5—C6	1.379 (14)	C15—H15A	0.9500
			
C2—Re1—C3	89.3 (4)	C7—C6—C5	119.2 (9)
C2—Re1—C1	89.3 (4)	C7—C6—H6A	120.4
C3—Re1—C1	89.0 (4)	C5—C6—H6A	120.4
C2—Re1—N1	96.0 (3)	C6—C7—C8	121.0 (9)
C3—Re1—N1	173.6 (3)	C6—C7—H7A	119.5
C1—Re1—N1	94.5 (3)	C8—C7—H7A	119.5
C2—Re1—N2	170.0 (3)	C7—C8—N1	120.6 (9)
C3—Re1—N2	99.1 (4)	C7—C8—C9	124.2 (9)
C1—Re1—N2	96.2 (3)	N1—C8—C9	115.2 (8)
N1—Re1—N2	75.3 (3)	N2—C9—C8	119.0 (9)
C2—Re1—Br1	92.1 (3)	N2—C9—H9A	120.5
C3—Re1—Br1	91.8 (3)	C8—C9—H9A	120.5
C1—Re1—Br1	178.4 (3)	C15—C10—C11	119.4 (9)
N1—Re1—Br1	84.6 (2)	C15—C10—N2	121.8 (8)
N2—Re1—Br1	82.3 (2)	C11—C10—N2	118.8 (8)
C4—N1—C8	117.7 (8)	C12—C11—C10	119.3 (9)
C4—N1—Re1	127.6 (7)	C12—C11—H11A	120.3
C8—N1—Re1	114.6 (6)	C10—C11—H11A	120.3
C9—N2—C10	115.7 (8)	C11—C12—C13	120.0 (9)
C9—N2—Re1	115.9 (7)	C11—C12—H12A	120.0
C10—N2—Re1	127.9 (6)	C13—C12—H12A	120.0
O1—C1—Re1	173.7 (9)	C14—C13—C12	121.0 (10)
O2—C2—Re1	178.9 (9)	C14—C13—Cl1	119.2 (8)
O3—C3—Re1	177.8 (9)	C12—C13—Cl1	119.9 (8)
N1—C4—C5	123.9 (10)	C15—C14—C13	119.9 (10)
N1—C4—H4A	118.1	C15—C14—H14A	120.0
C5—C4—H4A	118.1	C13—C14—H14A	120.0
C6—C5—C4	117.6 (10)	C14—C15—C10	120.4 (9)
C6—C5—H5A	121.2	C14—C15—H15A	119.8
C4—C5—H5A	121.2	C10—C15—H15A	119.8
